# LViT-Net: a domain generalization person re-identification model combining local semantics and multi-feature cross fusion

**DOI:** 10.1186/s42492-025-00190-1

**Published:** 2025-04-16

**Authors:** Xintong Hu, Peishun Liu, Xuefang Wang, Peiyao Wu, Ruichun Tang

**Affiliations:** 1 Faculty of Information Science and Engineering, Ocean University of China, Qingdao, Shandong 266100 China; 2 School of Mathematical Sciences, Ocean University of China, Qingdao, Shandong 266100 China

**Keywords:** Domain generalization, Person re-identification, Feature fusion, Semantic representation, Dual-branch network architecture

## Abstract

In the task of domain generalization person re-identification (ReID), pedestrian image features exhibit significant intra-class variability and inter-class similarity. Existing methods rely on a single feature extraction architecture and struggle to capture both global context and local spatial information, resulting in weaker generalization to unseen domains. To address this issue, an innovative domain generalization person ReID method–LViT-Net, which combines local semantics and multi-feature cross fusion, is proposed. LViT-Net adopts a dual-branch encoder with a parallel hierarchical structure to extract both local and global discriminative features. In the local branch, the local multi-scale feature fusion module is designed to fuse local feature units at different scales to ensure that the fine-grained local features at various levels are accurately captured, thereby enhancing the robustness of the features. In the global branch, the dual feature cross fusion module fuses local features and global semantic information, focusing on critical semantic information and enabling the mutual refinement and matching of local and global features. This allows the model to achieve a dynamic balance between detailed and holistic information, forming robust feature representations of pedestrians. Extensive experiments demonstrate the effectiveness of LViT-Net. In both single-source and multi-source comparison experiments, the proposed method outperforms existing state-of-the-art methods.

## Introduction

Person re-identification (ReID) aims to reliably retrieve and identify specific individuals across different viewpoints and conditions using non-overlapping cameras [1]. Owing to its practical significance in real-world scenarios, such as public security and video surveillance, person ReID has attracted widespread research attention. In recent years, using large-scale datasets and powerful computational capabilities, supervised [2–8] and semi-supervised [9–11] learning paradigms have achieved superior performance in specific target domains. However, when re-ID models are directly deployed in new domains, they often experience significant performance degradation and insufficient domain generalization capability [12]. Therefore, improving the robustness and generalization ability of models in cross-domain applications remains a critical challenge that must be addressed.

To address the aforementioned issues, related research [13–15] has begun to focus on exploring effective methods for domain generalization person ReID (DG-ReID) to improve model performance in new domains. These methods include domain-invariant feature learning [16, 17], meta-learning [18–20], and BN-based learning [1, 12]. Considering that convolutional neural networks (CNNs) have demonstrated outstanding performance in various image classification tasks, most previous studies adopted CNNs (e.g., ResNet-50 [21] and MobileNetV2 [22]) as backbone networks for pedestrian feature extraction. However, compared with supervised or semi-supervised settings, the decline in domain generalization performance is primarily due to variations in illumination, background, camera angles, and resolution between the source and target domains. These differences cause significant intraclass variability and interclass similarity in pedestrian images [12]. Therefore, it is essential to incorporate global semantic information to improve the robustness and generalization capability of the model. However, for CNN-based methods, the capture of global features is inherently limited by the fixed size of the convolutional kernel receptive field.

In recent years, due to its powerful sequence modeling ability and self-attention mechanism, Transformer [23] has gradually shifted from natural language processing tasks to the field of computer vision and achieved remarkable results. TransReID [24] introduced transformers into person ReID, and some works have explored the generalization capabilities of vision transformers (ViTs) [25]. TransMatcher [26] used hard attention for cross-matching in similarity computation, making it more efficient for image matching; however, it still employs CNNs as the primary feature extractor. Ni et al. [27] were the first to investigate the generalization capabilities of pure transformers in domain generalization for person ReID. Based on this, the team also addressed the issue of insufficient metric learning in multi-source domain general person ReID [28] to enhance style diversity and generalization ability using a style-aware hard negative sample sampling strategy and a dynamic style mixing strategy. All these methods employ a single-branch network structure for feature learning (either CNN-based or Transformer-based), failing to effectively leverage both local features and global semantic representations. Therefore, this study explores how to combine the advantages of CNNs and Transformer models through a dual-branch network structure, retaining local feature details while enhancing global semantic representations, to improve the generalization ability of pedestrian feature learning.

This study focuses on learning domain-invariant generalized pedestrian features and a dual-branch parallel feature extraction network structure called LViT-Net, is proposed. A parallel hierarchy of local and global feature blocks is designed to extract local and global features efficiently. In the local branch, the local multi-scale feature fusion (LMSF) module performs multi-scale fusion of local features. In the global branch, ViT-based global blocks are output in stages, with the pedestrian feature output at each stage serving as the global feature input for the dual feature cross fusion (DFCF) module. DFCF further fuses local and global semantic information. Experiments demonstrate that the proposed method outperforms mainstream approaches on person ReID datasets. For example, in the single-source protocol, under the Market1501$\rightarrow$CUHK03 setting, the proposed method achieves 26.3% and 26.4% for Rank 1 and mAP, respectively. In the multi-source protocol, under the M+D+C$\rightarrow$MS setting, the method reaches 48.7% and 23.3% in Rank 1 and mAP, respectively.

The contributions of this study are summarized as follows.

1. A novel network framework, LViT-Net, which efficiently captures local spatial features and global semantic representations using global blocks and local blocks, respectively, is proposed.
2. The LMSF module, which effectively fuses feature information at different scales, is introduced.
3. The DFCF module, which performs targeted cross fusion between global and local features, is proposed. This enhances the understanding of local details as well as global context.
4. The proposed LViT-Net model achieves outstanding performance in both single-source and multi-source experiments on the Market1501, DukeMTMC-reID, MSMT17, and CUHK03-NP datasets.

## Methods

### Overall framework

**Fig. 1:**
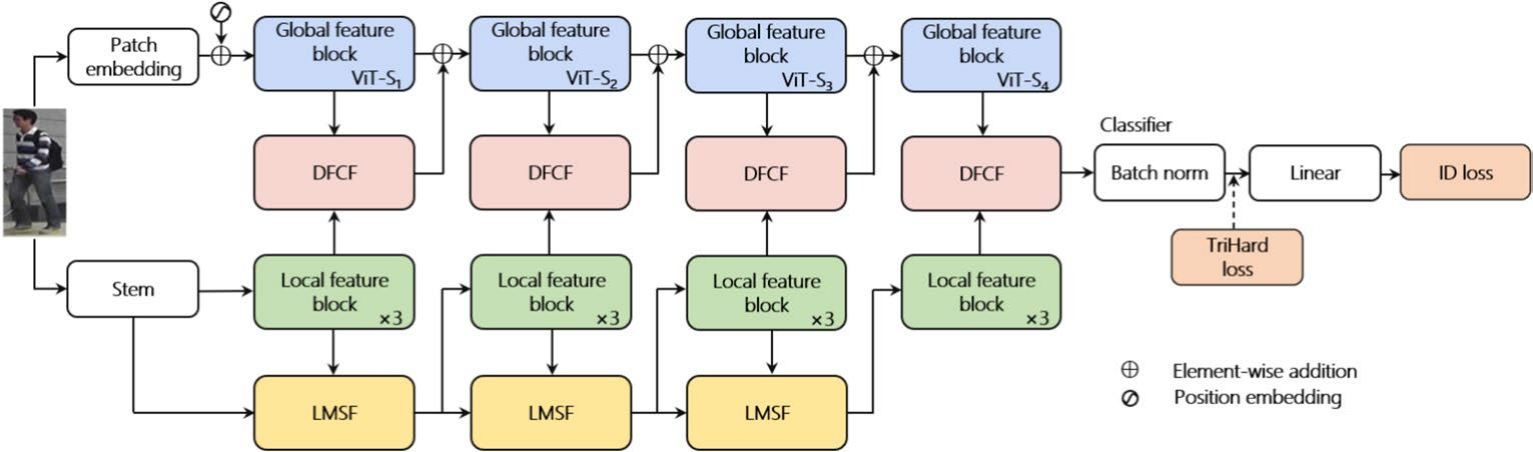
Overall framework of LViT-Net

To improve the accuracy of domain-generalized person ReID, a novel dual-branch person ReID model, LViT-Net, is proposed. LViT-Net aims to effectively obtain local spatial information and global semantic representations of pedestrian images at different scales. It adopts a parallel structure for staged feature fusion, as shown in Fig. 1.

The local branch is used to extract the local features of pedestrian images, whereas the global branch focuses on extracting global semantic representations. The initial stage of the local branch comprises a $4\times 4$ convolutional layer with a stride of four, followed by a Group Norm [29]. Different scales of local features are fused between local feature blocks using LMSF. The global branch utilizes a ViT structure, where the pedestrian image is non-overlappingly segmented by the patch embedding layer (e.g., each 16$\times$16 adjacent pixel forms a patch, which is then flattened along the channel direction, with learnable positional information and an additional learnable class token added). The parallel structure of LViT-Net maximizes the preservation of local features and global representations, with each branch consisting of four stages. The DFCF is used to fuse local features and global representations at each stage. Finally, the fused features are fed into a linear classifier with a Batch Norm for classification.

### Global feature block

**Fig. 2:**
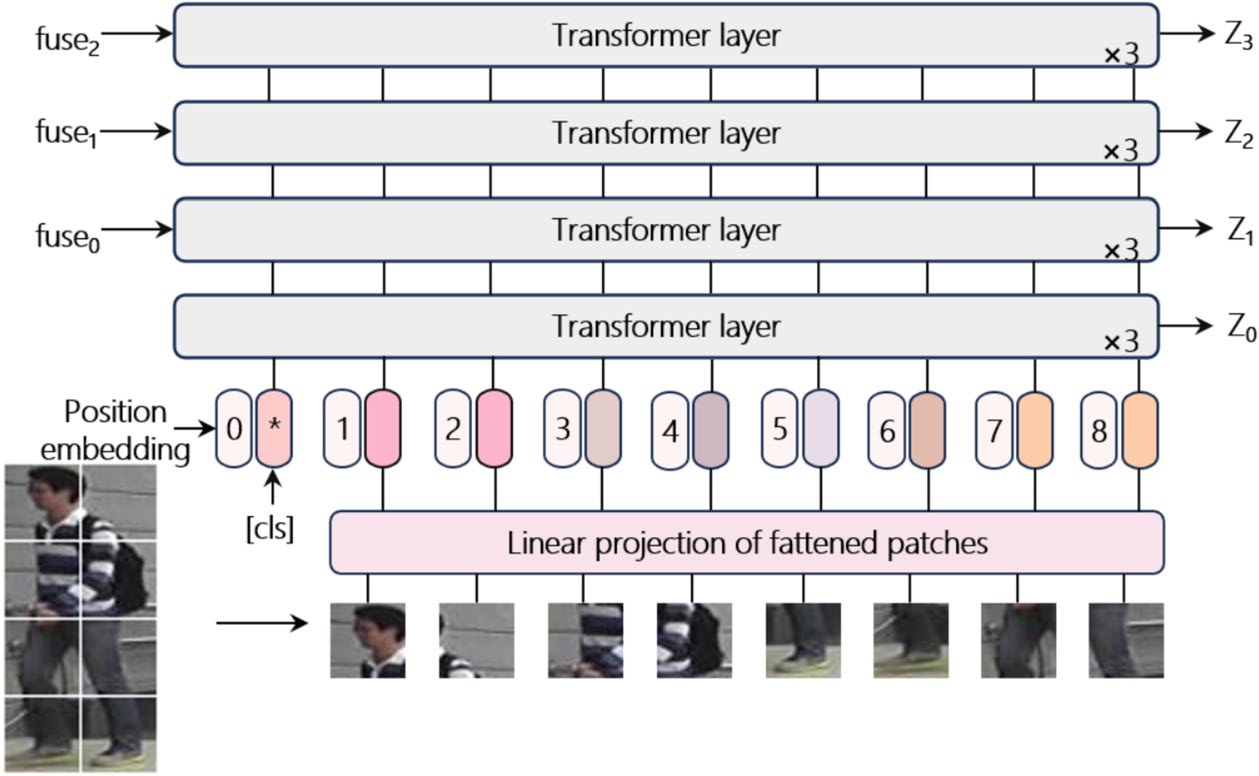
Structure of the global feature block

Because pedestrian images are captured under different conditions, they exhibit significant intraclass variation and interclass similarity, making it crucial to acquire global semantic information. The global branch is based on TransReID [24] using ViT as the feature extractor. Considering ViT-B/16 as an example, pedestrian images are passed through ViT for feature extraction, and the output features are saved every three layers, effectively dividing the entire ViT model into four stages. The output from each stage is used for subsequent feature fusion. The structure of the global feature block is shown in Fig. 2.

In each stage, the feature map are processed by multihead attention modules and a feedforward neural network to learn the global pedestrian features. Starting in the second stage, the fused features of the global and local characteristics from the previous stage are used as input features for the transformer encoder, as shown in Eq. 1.

1 $$\begin{aligned} & z_{0}=[x_{cls};x_{p}^{1}E;x_{p}^{2}E;...;x_{p}^{N}E]+E_{pos} \nonumber \\ & z_0=Transformer_1(z_0) \nonumber \\ & z_{k}^{^{\prime }}=Fusion(fuse_{k-1},z_{k}) \nonumber \\ & z_{k+1}=Transformer_{k+1}(z_{k}^{^{\prime }}) \end{aligned}$$

Here, $E_{pos}\in \mathbb {R}^{N\times d}$ represents the positional encoding, *E* represents the linear projection matrix, $Transformer_{1}$ denotes the transformer encoding from the previous three layers, *Fusion* represents the feature fusion operation, and $Transformer_{k+1}$ represents the transformer encoding at the current stage.

### Local feature block

**Fig. 3:**
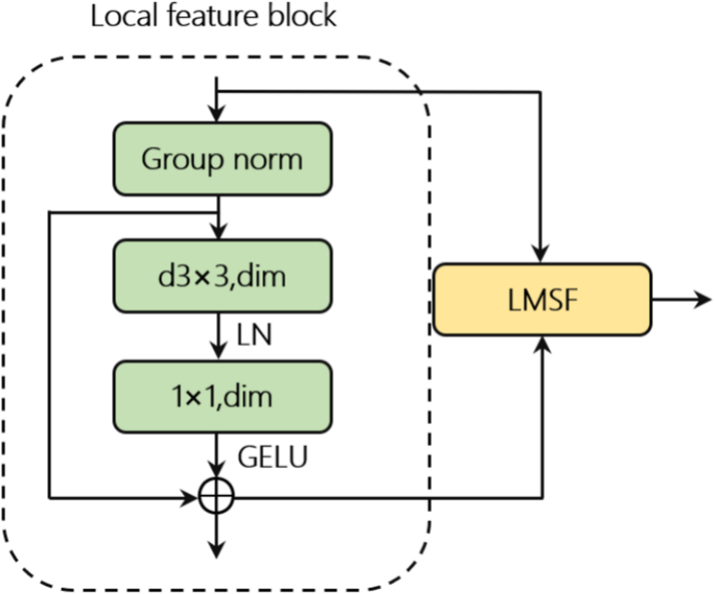
Structure of the local feature block

Local features of pedestrian images are also crucial for person ReID. As shown in Fig. 3, inspired by the concepts of ConvNeXt [30] and HiFuse [31], the local feature block employs depthwise convolution, utilizing the idea of grouped convolution, with the number of groups equal to the number of channels. The use of depthwise convolution effectively reduces the computational load and number of parameters while also improving the training efficiency.

After pedestrian feature extraction through depthwise separable convolutions, cross-channel information interaction is performed using a linear layer, utilizing layer normalization and the GELU activation function. Finally, the extracted local features are fed into the DFCF module. This process is represented by Eq. 2.

2 $$\begin{aligned} & X_{i-1}^{\prime }=DownSample(X_{i-1}) \nonumber \\ & X_{i}=f^{1\times 1}(LN(f^{3\times 3}(X_{i-1})))+X_{i-1}^{\prime } \end{aligned}$$

Here, $X_{i}$ represents the output features of the local feature block, $f^{3\times 3}$ denotes the depthwise convolution operation of $3\times 3$, and $f^{1\times 1}$ represents the pointwise convolution operation of $1\times 1$. The downsampling operation includes group normalization (group norm with 16 groups) and $1\times 1$ convolution.

In the local branch, different scales of feature information are captured through the local feature block. Utilizing information at different scales presents a challenge. Therefore, an LMSF, is proposed.

### Local multi-scale feature fusion

**Fig. 4:**
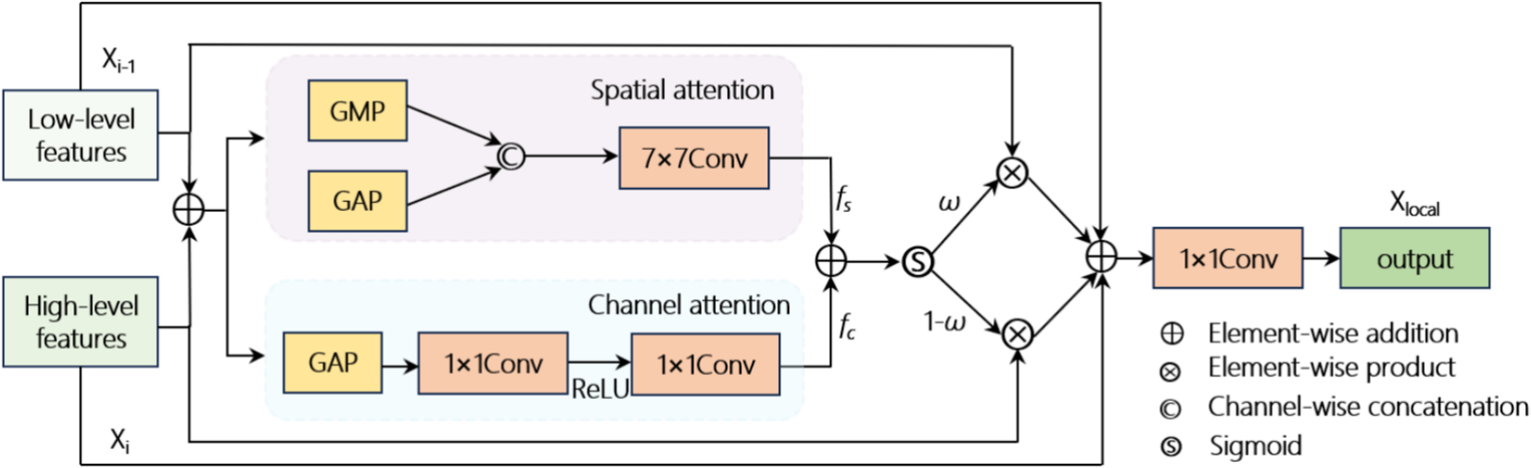
Detailed diagram of LMSF

The LMSF module primarily consists of channel and spatial attention (SA), effectively mixing channel and SA weights to ensure information interaction between low- and high-level pedestrian features. This enhances the local details, suppresses irrelevant features, and improves the semantic representation. The detailed process of LMSF is shown in Fig. 4. Let $X\in \mathbb {R}^{C\times H\times W},\Big (X=X_{i-1}+X_{i}\Big )$ represent the input features the goal of LMSF is to generate a channel-specific feature importance map with feature dimensions consistent with *X*.

First, calculate the corresponding $f_{C}$ and $f_{S}$ according to Eq. 3.

3 $$\begin{aligned} & f_{C}=f^{1\times 1}\biggl (\max \biggl (0,f^{1\times 1}\bigl (f_{\text {GAP}}^{\textrm{C}}\bigr )\biggr )\biggr ) \nonumber \\ & f_{S}=f^{7\times 7}\left( [f_{GAP}^{S},f_{GMP}^{S}]\right) \end{aligned}$$

Here, $\max (0,x)$ represents the ReLU activation function, $f^{k\times k}(\cdot )$ denotes a convolutional layer with a $k\times k$ kernel, $f_{GAP}^{c},f_{GAP}^{s},f_{GMP}^{s}$ represent global average pooling along the channel dimension, global average pooling along the spatial dimension, and global max pooling along the spatial dimension, respectively. During local feature fusion, low- and high-level features are combined using weighted summation, and residual connections are used to add input features, alleviating the vanishing gradient problem. This process is described in Eq. 4.

4 $$\begin{aligned} & \omega =\sigma (f_{C}+f_{S}) \nonumber \\ & X_{local}=f^{1\times 1}(\omega \cdot X_{i-1}+(1-\omega )\cdot X_{i}+X) \end{aligned}$$

Here, $\sigma$ represents the Sigmoid function, and $X_{local}$ denotes the fused output features.

### Dual feature cross fusion

LViT-Net adopts a parallel structure to extract pedestrian features from independent branches. The DFCF is designed to module to leverage the strengths of both local and global features, enhancing the expressive and discriminative ability of the model.

**Fig. 5:**
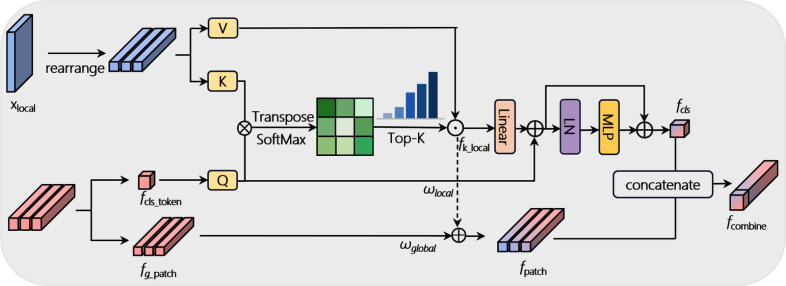
Detailed diagram of DFCF

As shown in Fig. 5, specifically, the features $X\in \mathbb {R}^{C\times H\times W}$ extracted from the local branch are first linearly transformed to match the dimension $X\in \mathbb {R}^{N\times d}$ of the global features (features extracted by ViT), where *N* represents the length of the feature sequence and *d* represents the feature dimension. Subsequently, the learned global feature cls_token ($f_{cls\_token}$) is extracted and projected as a query, generating the query vector *Q*. The adjusted local feature ($X_{local}$) is projected as a key and value, generating the key vector *K* and the value vector *V*, respectively. This process is described in Eq. 5.

5 $$\begin{aligned} & Q=Linear_{query}(f_{cls\_token}) \nonumber \\ & K=Linear_{key}(X_{local}) \nonumber \\ & V=Linear_{value}(X_{local}) \end{aligned}$$

Next, by calculating the dot product between *Q* and *K*, the attention scores are obtained and normalized using the SoftMax function. Attention scores are used to measure the correlation between each local feature and cls_token($f_{cls\_token}$). Based on the attention scores, the top-k local features ($f_{k\_local}$) with the highest scores are selected, and the corresponding features are extracted from the value vector. These features are then multiplied element-wise by *Q* to generate the fused cls_token ($f_{fuse\_cls}$). $f_{fuse\_cls}$ is further processed using linear transformation, layer normalization, and MLP to enhance its representation ability. This process is described in Eq. 6.

6 $$\begin{aligned} & f_{k\_local}=Topk(\frac{soft\max (QK^{T})}{\sqrt{d_{k}}}V) \nonumber \\ & f_{fuse\_cls}=Linear(f_{k\_local})+f_{cls\_token}\nonumber \\ & f_{fuse\_cls}^{^{\prime }}=MLP(LN(f_{fuse\_cls})\nonumber \\ & f_{cls}=f_{fuse\_cls}^{^{\prime }}+f_{fuse\_cls} \end{aligned}$$

Here, $f_{cls}$ denotes the fused cls_token generated by the DFCF.

Subsequently, the patch features ($f_{g\_patch}$) from the global features and the selected top-k local features ($f_{k\_local}$) are combined through weighted addition to generate the fused patch features($f_{patch}$). The weights for the global ($\omega _{global}$) and local features ($\omega _{local}$) are trainable parameters that ensure flexibility and adaptability during feature fusion. Finally, the fused cls_token and patch features are concatenated along the channel dimensions to generate the final feature representation ($f_{combine}$). This process is described in Eq. 7.

7 $$\begin{aligned} & f_{patch}=\omega _{global}\cdot f_{g\_patch}+\omega _{local}\cdot f_{k\_local} \nonumber \\ & f_{combine}=Concat(f_{cls},f_{patch}) \end{aligned}$$

The fused semantic features generated by DFCF are passed into the global branch for continued feature learning. In the global branch, the pedestrian features obtained after the final feature fusion are processed through a batch normalization layer and linear layer, and are used for the person ReID task.

### Loss function

The network is optimized by constructing ID loss and triplet loss for pedestrian features. $L_{ID}$ denotes the cross-entropy loss without label smoothing, as expressed in Eq. 8. For a triplet set $\{a,p,n\}$, the triplet loss with soft margin $L_{tri}$ is expressed in Eq. 8.

8 $$\begin{aligned} & L_{ID}=-\frac{1}{N}\sum \limits _{i=1}^{N}y_{i}\log (\hat{y_{i}}) \nonumber \\ & L_{tri}=\log [1+\exp (\left\| f_{a}-f_{p}\right\| _{2}^{2}-\left\| f_{a}-f_{n}\right\| _{2}^{2})] \end{aligned}$$

In the ID loss, *N* represents the total number of samples, $y_{i}$ denotes the ground truth label of the *i*-th sample, where the value is either 0 or 1, indicating whether it corresponds to the correct identity, and $\hat{y_{i}}$ represents the predicted label of the *i*-th sample. The triplet loss is calculated using the anchor features($f_{a}$), positive sample features($f_{p}$), and negative sample features($f_{n}$); the Euclidean distance used to measure the similarity between samples.

Finally, the overall loss is $L=\lambda _{ID}L_{ID}+\lambda _{tri}L_{tri}$.

## Results

### Datasets and evaluation metrics

Experiments are conducted on four large-scale person ReID datasets: Market1501 [32], DukeMTMC-reID [33], MSMT17 [10], and CUHK03-NP [34], as listed in Table 1. For convenience, the datasets are abbreviated as M, D, MS, and C, respectively. To evaluate the generalization ability of the model, both single-source [35] and multi-source protocols [36] are used. In the single-source protocol, the model is trained using one of the aforementioned datasets (training set only) and tested on another (testing set only). In the multi-source protocol, one domain from M + D + C + MS is selected for testing (testing set only) and the remaining domains are used for training (training set only), as shown in Table 2.

**Table 1:** Statistics of person ReID datasets

Dataset	ID	Image	Camera	Train	Query	Gallery
Market1501 [32]	1501	32,217	6	12,936	3368	15,913
DukeMTMC-reID [33]	1812	36,411	8	16,522	2228	17,661
MSMT17 [10]	4101	126,441	15	30,248	11,659	82,461
CUHK03-NP [34]	1467	28,192	2	7365	1400	5332

**Table 2:** Evaluation protocols

Protocol	Training set	Testing set
Multi-source protocol	M + D + C	MS
	MS + M + C	D
	MS + M + D	C
	MS + D + C	M
Single-source protocol	Train-Market	D, C, MS
	Train-Duke	M, C, MS
	Train-MSMT	M, D, C

In terms of evaluation metrics, the performance is quantitatively evaluated by mean average precision (mAP) and cumulative matching characteristic (CMC) at Rank-1 (R1). The calculation methods are shown in Eqs. 9 and 10.

9 $$\begin{aligned} cmc(N)=\sum \limits _{n=1}^Nr(n) \end{aligned}$$

Here, *N* denotes the number of queries, and *r*(*n*) represents Rank-n.

10 $$\begin{aligned} AP=\frac{1}{2}\sum \limits _{i=2}^M((recall_i-recall_{i-1})(precise_i+precise_{i-1})) \end{aligned}$$

Here, *M* denotes the number of identities in the test set. The final mAP is obtained by summing the average precision of all query batches and then averaging the results.

### Experimental details

In the global branch, the structure of ViT is not altered; therefore, the ViT-based model pretrained on ImageNet with a patch size of 16 ( ViT-B/16) is used as the backbone network for the global branch. In this experiments, the settings are consistent with those in ref. [27] , we set the batch size to 64 and resize all input images to 224 $\times$ 224 to meet the input requirements of the backbone network. During training, the images are normalized using a mean of [0.5, 0.5, 0.5] and a standard deviation of [0.5, 0.5, 0.5], scaling pixel values to the range of [−1, 1], thereby improving the stability of data distribution and accelerating model convergence. Additionally, we apply Local Grayscale Transformation [37] with a 50% probability, converting parts of the image to grayscale to simulate low-light or monochromatic conditions. Random horizontal flipping is also enabled, with a flip probability of 50%, further enhancing the generalization ability of the model in diverse real-world scenarios. The model is optimized using an SGD optimizer with a weighted decay coefficient of $10^{-4}$. The learning rate starts from 0 and linearly increases to $10^{-3}$ over the first 10 epochs, and then gradually decays over the next 50 epochs. The entire training process consists of 60 epochs.

### Experimental results

(1) **Single-source DG ReID** To verify the performance of the proposed model, the framework is evaluated using the single-source DG ReID benchmark. Specifically, Market-train, Duke-train, and MSMT-train are used as training sets, and Market-test, Duke-test, MSMT-test, and CUHK03-test as test sets.

The experimental results are shown in Tables 3 and 4. The results are highlighted in bold; the best results are highlighted in red, and the second-best results are highlighted in blue. For the CUHK03 dataset, the new protocol, CUHK03-NP, which is more challenging than the original CUHK03 protocol, is adopted. LViT-Net demonstrates superior performance compared to state-of-the-art models in most settings. When trained on the market dataset, the proposed model achieves R1 and mAP values of 68.2% and 49.1%, respectively, when tested on Duke; 42.4% and 18.6%, respectively, when tested on MSMT; and 26.3% and 26.2%, respectively, when tested on CUHK03-NP, which are 0.9% and 0.2% higher than those of the ViT-based model PAT [27]. When trained on the Duke dataset, the proposed model achieves R1 and mAP values of 71.4% and 45.7%, respectively, when tested on Market. R1 and mAP are 2.2% and 1.2% higher than PAT when tested on MSMT, and 19.3% and 19.2%, respectively, when tested on CUHK03-NP. When trained on the MSMT dataset, the proposed model achieved R1 and mAP values of 79.0% and 49.6%, respectively when tested on Market, and outperforms PAT by 0.7% and 1.4%, respectively. When tested on the Duke dataset, R1 and mAP reaches 72.1% and 53.8%, respectively. These results demonstrate the superiority of the proposed model.

**Table 3:**
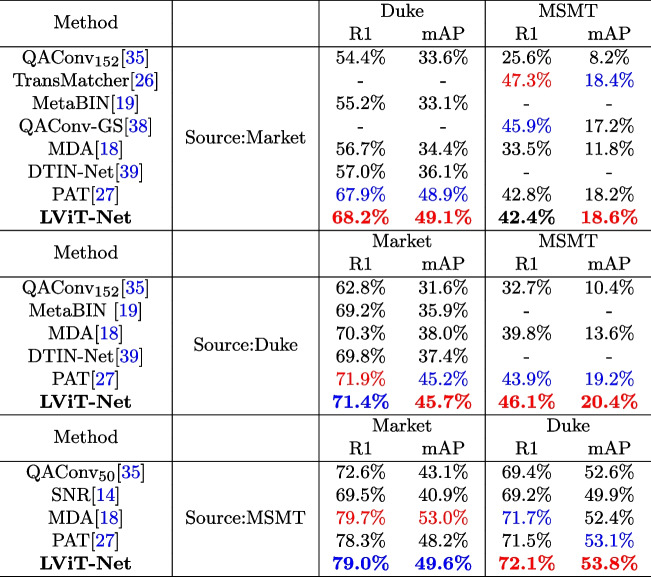
Performance comparison between LViT-Net and the SOTA methods for single-source DG ReID on Market1501, DukeMTMC-reID, and MSMT17 [14, 18, 19, 26, 27, 35, 38, 39]

**Table 4:**
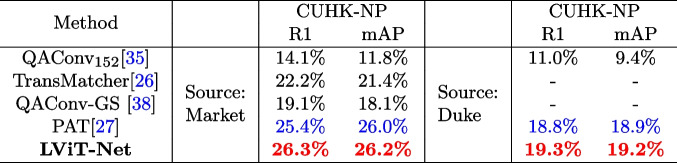
Performance comparison between LViT-Net and the SOTA methods on single-source DG ReID under M$\rightarrow$C and D$\rightarrow$C settings [26, 27, 35, 38]

(2) ** Multi-source DG ReID** To further verify the generalization ability of the proposed model, experiments are conducted using a multisource protocol. The multi-source evaluation protocol shown in Table 2 is followed. Specifically, three datasets from Market1501 (M), DukeMTMC-reID (D), MSMT17 (MS), and CUHK03-NP (C) are used as the source domains, with the remaining dataset as the target domain.

**Fig. 6:**
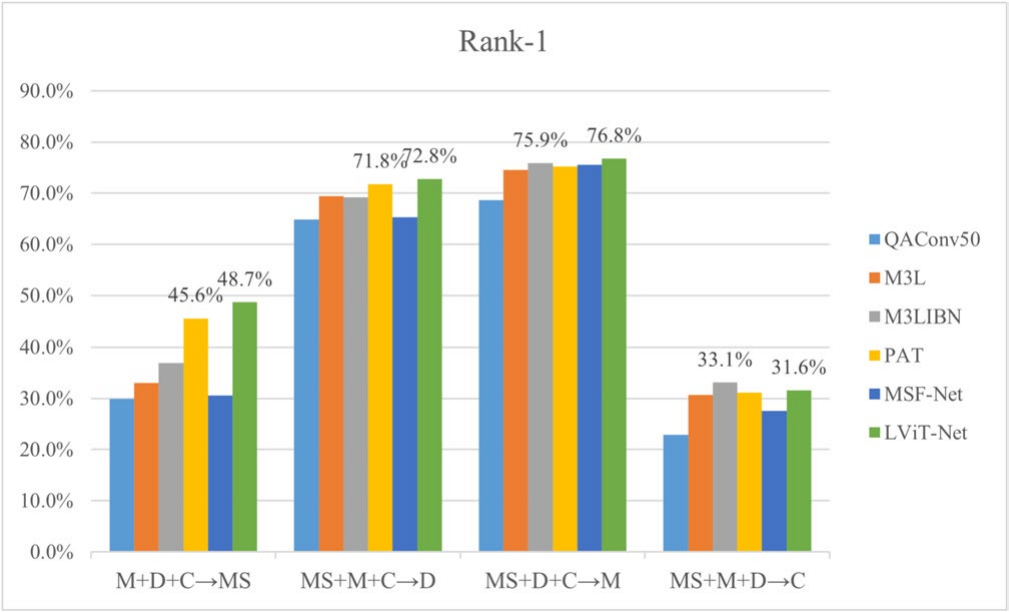
Comparison of Rank-1 with the latest DG models across four large-scale person ReID benchmarks

**Fig. 7:**
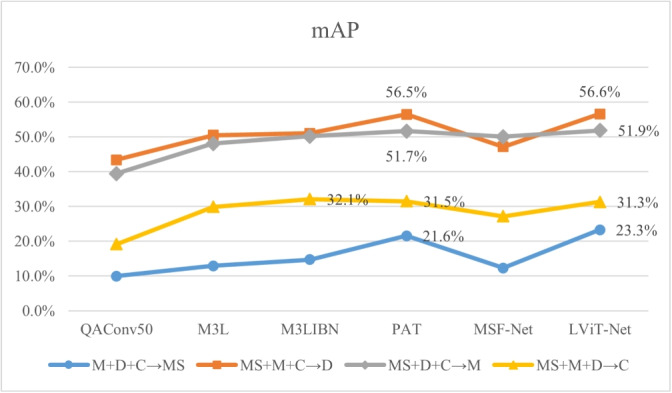
Comparison of mAP with the latest DG models across four large-scale person ReID benchmarks

As shown in Figs. 6 and 7, under the M + D + C$\rightarrow$MS setting, the R1 and mAP of the proposed model are 3.1% and 1.7% higher than the SOTA model (PAT), respectively; under the MS + M + C$\rightarrow$D setting, the R1 of our model reaches 72.8%, which is 1.0% higher than PAT. Under the MS + D + C$\rightarrow$M setting, the R1 and mAP of the proposed model are 0.9% and 0.2% higher than PAT, respectively. In addition, under the MS + M + D$\rightarrow$C setting, the proposed model still shows similar performance compared to PAT.

### Ablation studies

This subsection includes ablation studies to investigate the effectiveness of the proposed approach, including the effectiveness of LMSF, DFCF, and dual-branch network, as well as the visualization of the model. We also consider the impact of weather and environmental conditions, as well as performance variations under noisy conditions.

1. Is a local multi-scale feature fusion necessary?

**Fig. 8:**
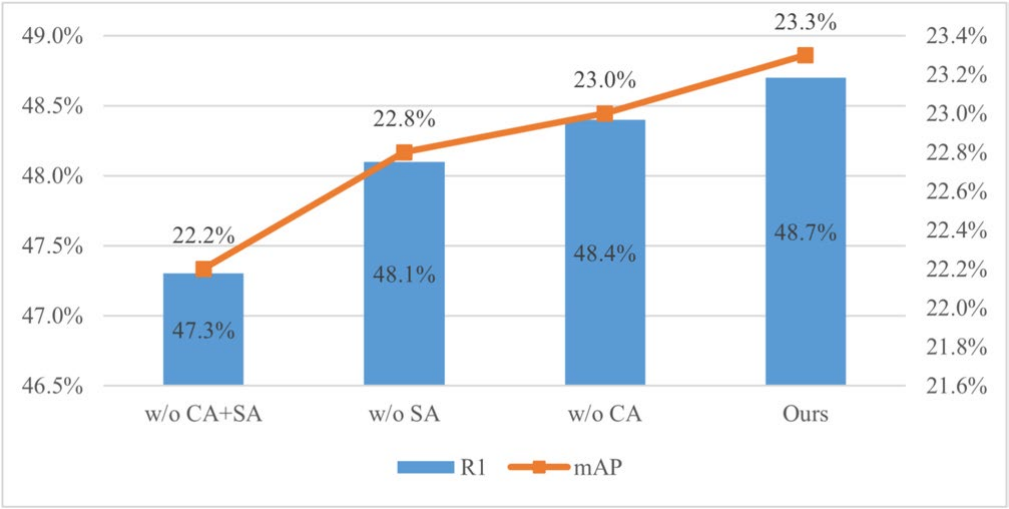
Impact of using LMSF

LMSF mainly includes channel attention (CA) and SA. Experiments are conducted under the M + D + C$\rightarrow$MS setting to compare the results of (1) w/o CA + SA, (2) w/o SA, and (3) w/o CA with those of LViT-Net. The experimental results shown in Fig. 8 reveal that when the local branch does not include CA + SA, both R1 and mAP decrease significantly. When using only one type of attention (either CA or SA), the performance of LViT-Net decreases to some extent; however, the impact remains relatively minor. Overall, LMSF combining channel and SA, can effectively improve the performance of the model.

(2) How many times should dual feature cross fusion be performed?

Experiments are conducted with DFCF applied 1, 2, 3, 4, 5, and 6 times to explore the optimal number of fusion iterations.

**Fig. 9:**
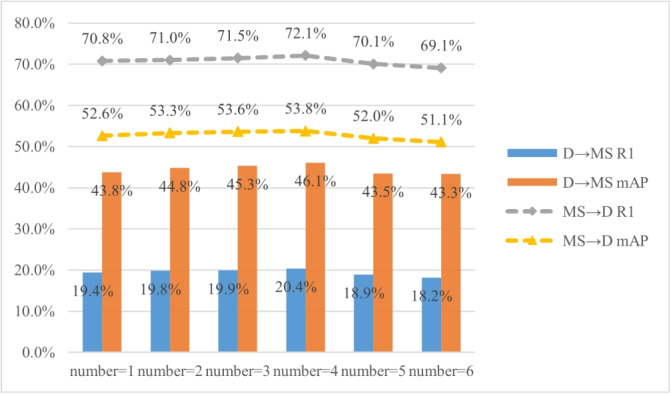
Ablation experiment results of the number of DFCF

As shown in Fig. 9, the experimental results indicate that with an increase in the number of dual feature cross fusion, both R1 and mAP of the model improved. However, when the number of fusions exceeds 5, the performance of model begins to decline. When the number of DFCF is 4, under the D$\rightarrow$MS setting, the R1 increases to 20.4%, and the mAP increases to 46.1%. Under the MS$\rightarrow$D setting, the R1 increases to 72.1%, and the mAP increases to 53.8%. This demonstrates that multiple dual feature cross fusion can significantly enhance the performance of the model, with the best results achieved when the fusion is applied four times.

(3) The effectiveness of the dual-branch network.

To verify the effectiveness of the dual-branch network, experiments are conducted on the global branch and local branch separately under the D$\rightarrow$MS setting, comparing recognition results of their pedestrian images, as shown in Table 5.

**Table 5:** Ablation study of the dual-branch network structure

Method	D$\varvec{\rightarrow }$MS	
	R1	mAP
w/o global	41.2%	17.8%
w/o local	44.1%	18.1%
Ours	46.1%	20.4%

As shown in Table 5, the dual-branch network structure of LViT-Net improves the accuracy of pedestrian image recognition.

(4) Visualization

**Fig. 10:**
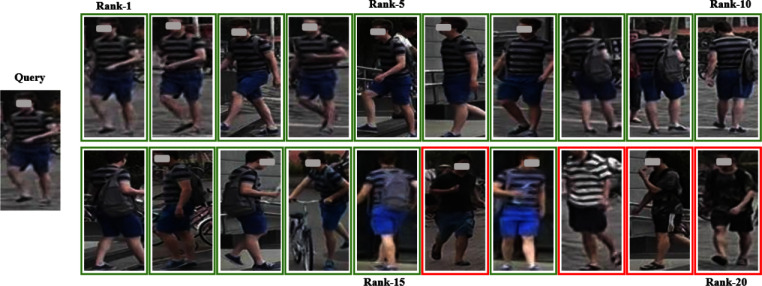
Recognition results of LViT-Net on the Market-1501

**Fig. 11:**
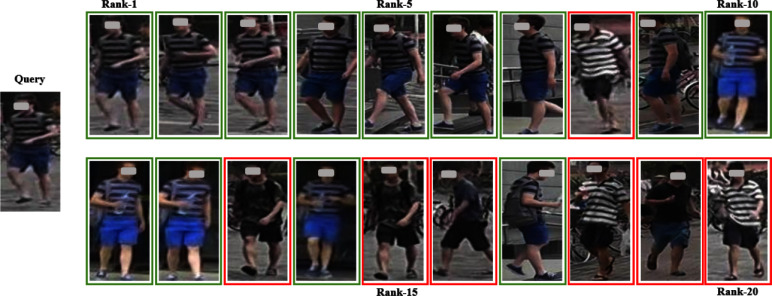
Recognition results of PAT on the Market-1501

**Fig. 12:**
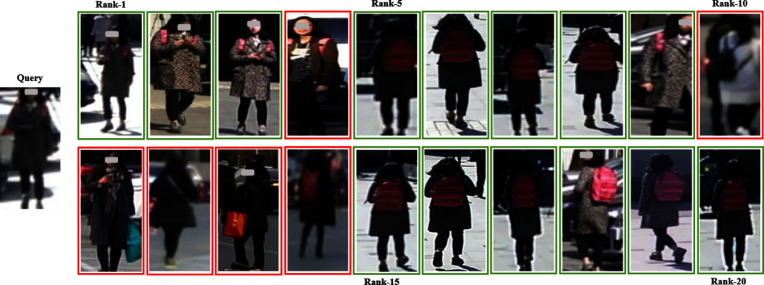
Recognition results of LViT-Net on the MSMT

**Fig. 13:**
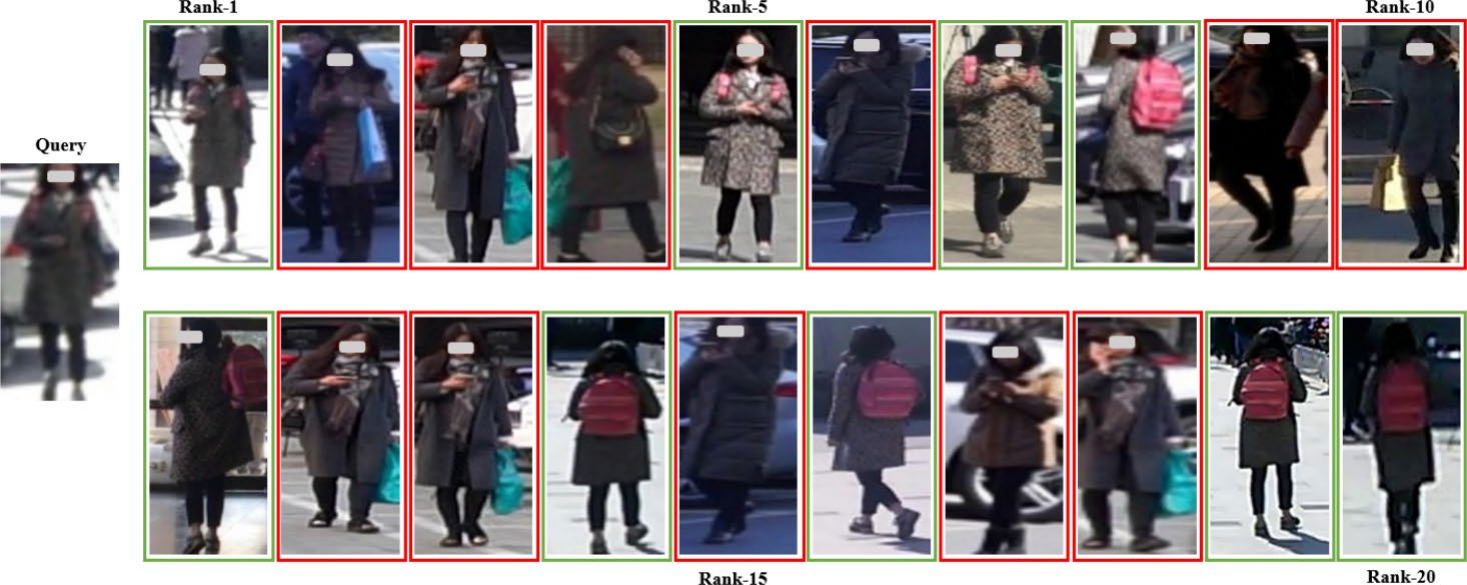
Recognition results of PAT on the MSMT

To more intuitively compare the performance of the model, the recognition results on the Market-1501 and MSMT datasets are visualized using an optimal weight in a single-source setting and a multi-source setting, and compared with the SOTA model PAT [27]. The visualization results are shown in Figs. 10, 11, 12, and 13. LViT-Net has a good recognition effect and robustness, and its recognition accuracy is higher than that of PAT. The top 20 matching results are selected for comparison and the retrieval results are arranged in that order. The green solid line indicates that the pedestrian in the image has the same ID as the pedestrian to be queried and the red solid line indicates that the pedestrian in the image has a different ID than the pedestrian to be queried.

**Fig. 14:**
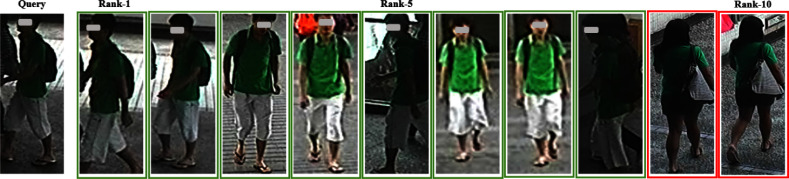
Recognition results of LViT-Net on the CUHK03

**Fig. 15:**
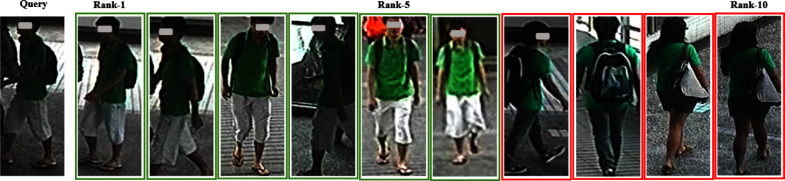
Recognition results of PAT on the CUHK03

Because the new CUHK03 protocol imposes strict divisions on the dataset, with each person ID having at most ten images, this may result in insufficient data diversity. Consequently, the generalization performance of CUHK03, as shown in Tables 3 and 4, is less favorable than that of the other datasets. Visual analysis of the CUHK03 dataset is performed using the best weights from the single-source setting and the top 10 matching results compared. As shown in Figs. 14 and 15, the retrieval results are arranged in order, with green solid lines indicating that the person in the image has the same ID as the query and red solid lines indicating that the person in the image has a different ID from the query. Compared with the recognition results of PAT, LViT-Net demonstrates higher recognition accuracy.

**Fig. 16:**
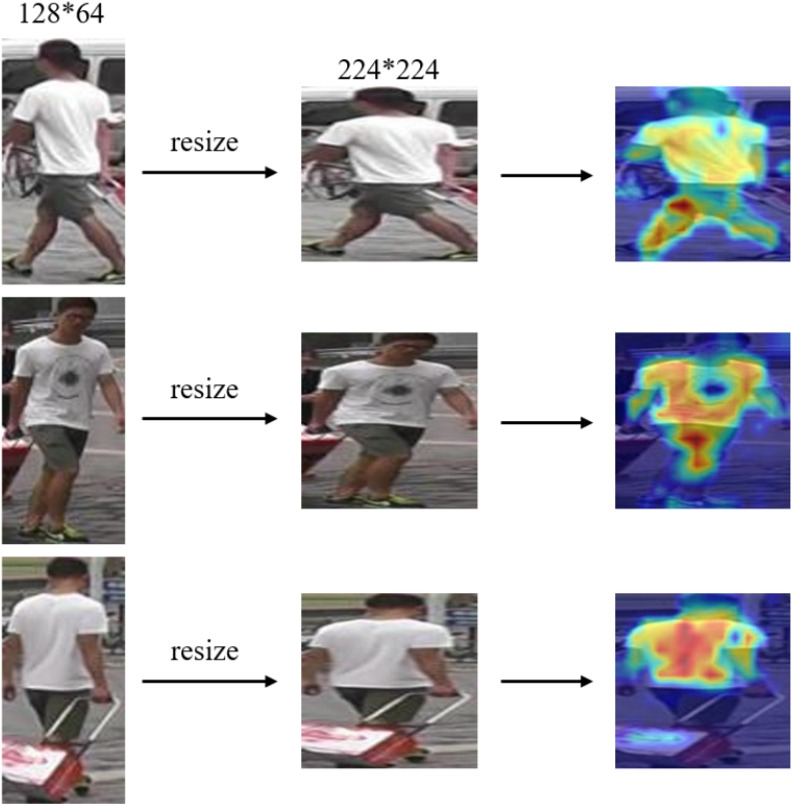
Heatmap of feature extraction visualization based on Grad-CAM

In addition, inspired by the visualization analysis of feature extraction in refs. [24, 27, 40], Grad-CAM [41] is employed to generate heatmaps for the final features extracted by the model. The feature extraction of the same person using different cameras and viewpoints is visualized and analyzed, as shown in Fig. 16. In the figure, the colors range from blue to red and represent the feature weight values from lowest to highest. The proposed model effectively learns pedestrian features, with a more complete and accurate focus on the pedestrian target.

(5) The impact of weather and environmental conditions.

**Fig. 17:**
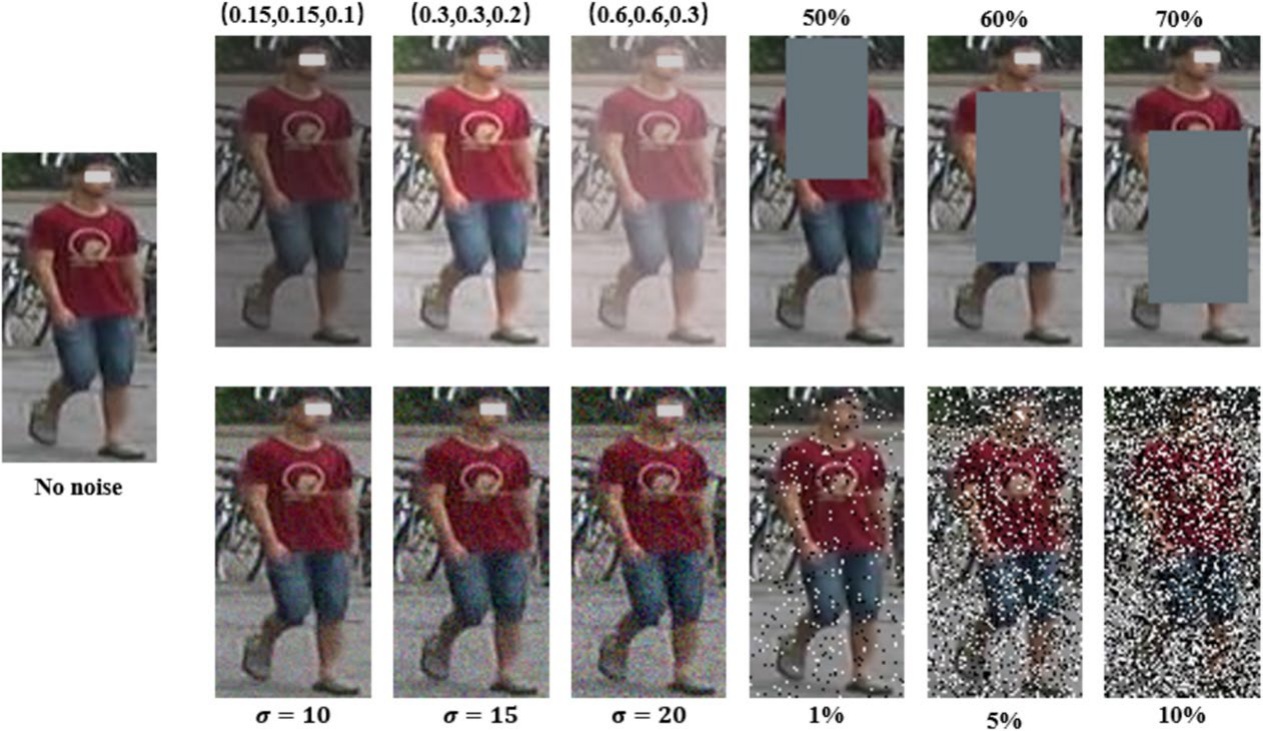
The pedestrian images considering weather, environmental conditions and noise

Considering the impact of weather and environmental conditions, we conduct experiments simulating real-world scenarios under both single-source and multi-source protocols. We introduce lighting variations (ColorJitter) and random occlusion (RandomErasing) to compare their impact on performance. The images after the transformations are shown in Fig. 17. The experimental results are shown in Table 6.

**Table 6:** Ablation study considering the impact of weather and environmental conditions

Method	MS + M + C$\varvec{\rightarrow }$D		M$\varvec{\rightarrow }$C	
	R1	mAP	R1	mAP
NO	72.8%	56.6%	26.3%	26.2%
CJ (0.15,0.15,0.1)	72.1%	55.5%	23.7%	24.0%
CJ (0.3,0.3,0.2)	72.5%	55.9%	22.6%	24.0%
CJ (0.6,0.6,0.3)	72.1%	56.3%	23.6%	24.8%
REA (50%)	72.5%	56.5%	24.4%	25.3%
REA (60%)	72.4%	56.2%	23.1%	24.8%
REA (70%)	72.2%	56.3%	23.9%	25.0%

We set the lighting variations to (0.15, 0.15, 0.1), (0.3, 0.3, 0.2), and (0.6, 0.6, 0.3), where each parameter represents brightness, contrast, and saturation, respectively. The random erasing probabilities are set to 50%, 60%, and 70%. Experimental results show that lighting variations and random erasing slightly reduce the performance of the model. This may be because the simulation process causes the generated data to deviate from the real distribution, making it difficult for the model to learn effective and critical discriminative information. Furthermore, we observe that increasing lighting variation and random erasing probability does not consistently degrade performance, indicating that LViT-Net demonstrates strong feature extraction capability and robustness.

(6) The impact of noise.

To demonstrate the impact of noise on ReID, we conduct noise experiments on both LViT-Net and PAT [27]. Pedestrian images processed with different levels of Gaussian noise and salt-and-pepper noise are shown in Fig. 17. We compare and analyze their performance, as shown in Table 7.

**Table 7:** Ablation study on the impact of noise

Noise	PSNR (dB)	M$\varvec{\rightarrow }$C			
		LViT-Net		PAT	
		R1	mAP	R1	mAP
NO noise	$\infty$	26.3%	26.2%	25.4%	26.0%
Gaussian noise ($\sigma =10$)	28.13	23.4%	24.5%	22.5%	23.6%
Gaussian noise ($\sigma =15$)	24.52	22.7%	23.8%	22.6%	23.3%
Gaussian noise ($\sigma =20$)	22.11	22.2%	23.1%	21.8%	22.3%
Salt-and-pepper noise (1%)	20.00	21.8%	22.7%	22.2%	22.3%
Salt-and-pepper noise (5%)	13.01	21.4%	22.0%	20.6%	21.3%
Salt-and-pepper noise (10%)	10.00	20.4%	21.3%	20.1%	20.5%

The experimental results demonstrate the performance comparison between LViT-Net and PAT under different noise conditions. As the standard deviation of Gaussian noise or the proportion of salt-and-pepper noise increases, the Rank-1 accuracy and mAP of both models decrease significantly. LViT-Net consistently outperforms PAT under all noise conditions, indicating better robustness to noise interference. Moreover, salt-and-pepper noise has a more pronounced impact on performance compared to Gaussian noise, especially at higher noise levels. The lower the PSNR, the greater the performance degradation, confirming the significant impact of noise on person ReID.

## Discussion

The feasibility and effectiveness of the model is verified using single- and multi-source protocols. According to Tables 3 and 4 and Figs. 6 and 7, LViT-Net is compared with the latest DG-ReID models. The proposed method outperforms existing SOTA models in terms of Rank1 and mAP. For instance, under the M + D + C$\rightarrow$MS setting, the R1 and mAP of LViT-Net are 3.1% and 1.7% higher than PAT [27], respectively. Although the performance of the model on the CUHK03 dataset is still lower than that on the other datasets, the proposed method outperforms the SOTA model. Simultaneously, matching visualizations are conducted on the CUHK03 dataset, as shown in Figs. 14 and 15. The results indicate that the matching accuracy of LViT-Net is also better than PAT. In the future, the focus will be on further optimizing the generalization performance of this dataset.

Through experiments and visualization results, it has been observed that even with the best weights under single-source and multi-source protocols, the model cannot perfectly match the same pedestrian. Taking the Market1501 dataset as an example, the matching error rate of LViT-Net is 20%, while that of PAT is 35%, indicating that LViT-Net performs better than PAT but still exhibits certain errors. The main reasons lie in the significant differences between datasets, including variations in viewpoints, backgrounds, and environments. For example, different viewpoints may lead to inconsistent feature extraction, complex background interference reduces matching accuracy, and variations in clothing and posture further increase the difficulty. Moreover, differences in resolution and image quality limit the ability to capture fine details, and domain bias between datasets weakens the cross-domain generalization performance of the model. Future work will focus on further research in these areas.

LViT-Net adopts a parallel structure utilizing both local and global feature encoders to simultaneously extract local and global features from pedestrian images, thereby enriching the diversity of pedestrian image features. To fully leverage the local pedestrian features at different scales, the LMSF module is used between local feature blocks to perform LMSF. This plug-and-play module integrates both channel and SA. As shown in Fig. 8, the effectiveness of the LMSF module in improving the performance of the model and addressing the issue of missing local feature extractions in PAT [27] has been proven. To fully exploit the complementary relationship and correlation between local and global features, the DFCF module is used between the local and global branches to fuse local features with global semantic information. As shown in Fig. 9, the impact of the number of DFCF modules used on the model performance is verified. Under the D$\rightarrow$MS setting, LViT-Net with four DFCF modules has Rank1 and mAP 2.2% and 1.2% higher than PAT, respectively. This demonstrates that the DFCF module can effectively fuse local and global features, thereby generating domain-invariant pedestrian features for person ReID.

## Conclusions

In this study, a domain generalization person ReID method based on dual-branch feature fusion is proposed to address the problem of insufficient extraction of local features and global semantic information in a domain generalization person ReID task. Local and global feature blocks are designed to extract local features and global representations in parallel, respectively. Effective fusion between low- and high-level local features through the LMSF is achieved. In addition, the DFCF is used to selectively fuse local features and global representations to accurately match related features and further improve the generalization ability of the model. Experimental results show that LViT-Net performs better than mainstream domain generalization person ReID methods in recent years.

However, despite these advancements, there are still some limitations in the proposed model that need to be considered. As the global branch of the model relies on the architecture of the vision transformer, the computational cost is relatively high, which may affect the recognition speed in real-world applications. Additionally, because standard person ReID datasets are used, the performance of the model might have been impacted on datasets with occlusions. In the future, further experiments on occluded datasets will be conducted. Furthermore, cases in which relying solely on images cannot achieve accurate identification will be explored, and the generalization performance of text-to-image cross-modal person ReID will be studied.

## Data Availability

No new data were created. Data are available in a publicly accessible repository. The data presented in this study are openly available in refs. [10, 32–34].

## Abbreviations

Person ReID: Person re-identification
DG-ReID: Domain generalization person re-identification
CNN: Convolutional neural network
ViT: Vision transformer
LMSF: Local multi-scale feature fusion
DFCF: Dual feature cross fusion
M: Market1501
D: DukeMTMC-reID
MS: MSMT17
C: CUHK03-NP
mAP: Mean average precision
CMC: Cumulative matching characteristic
R1: Rank-1
CA: Channel attention
SA: Spatial attention
